# Health-Related Quality of Life and Psychological Burden Among and Beyond Children and Adolescents With Type 1 Diabetes: A Family Perspective

**DOI:** 10.7759/cureus.81744

**Published:** 2025-04-05

**Authors:** Stefanos Karakolias, Anastasia Iliopoulou

**Affiliations:** 1 Post-Graduate Program in Health Units Management, Hellenic Open University, Patras, GRC

**Keywords:** children and adolescents, diabetes type 1, health-related quality of life (hrqol), psychological burden, parents

## Abstract

Introduction

Type 1 diabetes in children and adolescents is a challenging situation for everyone involved. Given that previous research has focused primarily on patients, the impact on their family members is relatively underexplored. In this context, we aimed to evaluate the health-related quality of life (HRQoL) and psychosocial burden of both minor patients and their families.

Methods

This study involved 151 children/adolescents with type 1 diabetes and family members of such patients, both living in Greece. We applied a combined e-questionnaire including demographic and clinical data, psychological burden data as reflected by the Depression, Anxiety and Stress Scale - 21 Items (DASS-21), and HRQoL data as reflected by the 36-Item Short Form Survey (SF-36). Statistical analysis included descriptive statistics and inferential analysis.

Results

Regarding psychological burden, our study revealed mild to normal depression levels (10.6 ± 5.6), mild to moderate stress levels (16.2 ± 5.8), and moderate anxiety levels (10.6 ± 5.6); therefore, anxiety seems to prevail among psychological burden conditions. Regarding HRQoL, moderate levels of overall physical health (50.5 ± 12.7) and moderate to low levels of overall mental health (44.5 ± 13.2) were observed. Stress, anxiety, depression, and HRQoL tend to be significantly influenced by various demographic and clinical attributes. As expected, an inverse correlation between HRQoL and psychological burden was observed.

Conclusion

The treatment of pediatric type 1 diabetes seems to be a family matter, given that family members are deeply involved in the general run of things. To enhance HRQoL and resilience against psychological burden for minor patients, both the patients and their caregivers/parents should be supported accordingly.

## Introduction

Type 1 diabetes mellitus is a chronic inflammatory autoimmune disease caused by the destruction of pancreatic beta cells, leading to lifelong dependence on external insulin [1]. It is one of the most common diseases globally and is a particularly frequent chronic illness among children and adolescents, with an increasing incidence rate [2,3]. The treatment of type 1 diabetes mellitus relies on disease management and maintaining blood glucose levels as close to normal as possible [1]. This requires a strict daily self-care regimen, including regular monitoring of blood glucose levels, insulin administration, regular physical exercise, and proper dietary management [4].

Owing to the challenging long-term treatment, managing pediatric diabetes can be particularly challenging for both patients and their caregivers/parents [2,5]. Anxiety and depression disorders are quite common, and the challenges parents face in enforcing a strict diabetes care regimen can be overwhelming [4]. These burdens often have a significant impact on the physical, mental, and emotional well-being and the quality of life of children/adolescents suffering from the disease and their caregivers/parents [3,6].

Although the effects of diabetes on the health-related quality of life (HRQoL) and mental state of adult patients have been extensively researched, there is limited research on pediatric patients. Furthermore, most existing studies focus on patients themselves rather than their parents and other family members, who are also significantly burdened by diabetes care. In Greece, only a few relevant studies have been identified in recent years, highlighting the need for further research on the Greek population. Therefore, the primary purpose of this study was to investigate the impact of pediatric and adolescent type 1 diabetes on the HRQoL and psychological burden of both patients and their families in Greece.

## Materials and methods

Study design and participants

From the outset, we intended to conduct a cross-sectional study which would be twofold regarding research topics and research subjects. We planned to combine HRQoL with psychological burden, such as stress, anxiety, and depression. Moreover, we aimed to investigate these aspects among both children and adolescents with type 1 diabetes and their family members. Thus, our primary purpose was to assess the HRQoL, stress, anxiety, and depression levels of these individuals. However, two individual research objectives emerged: first, to examine any significant differences in HRQoL, stress, anxiety, and depression based on demographic or clinical attributes, and second, to explore any correlations between stress, anxiety, depression, and HRQoL scores.

To achieve our research objectives, we used a three-part electronic questionnaire. Part 1 consisted of questions regarding the demographic and clinical information of the participants (see Tables 19, 20 in the Appendices). Part 2 focused on assessing the levels of stress, anxiety, and depression, and Part 3 was dedicated to HRQoL.

The sample for this study comprised 151 respondents residing in Greece, among whom 72 were children/adolescents with type 1 diabetes and 79 were family members. There were no individual participants belonging to the same family. Thus, this study focused on 151 individual patients, but in 79 cases, it captured their family perspective. The survey items were identical for both types of participants. We applied quota sampling aiming to achieve rational spread across the target population based on their demographics (e.g., age and gender). The inclusion criteria for patients were a diagnosis of type 1 diabetes and age (≤19 years). The inclusion criterion for family members was active participation in the care of a patient meeting the inclusion criteria for patients at the time of the survey (March-April 2024). The exclusion criterion for both types of participants was being unable to give informed consent. The survey was conducted remotely by distributing an e-questionnaire. Participation was voluntary and anonymous in nature. Before completing the questionnaire, participants were provided with a brief informational note outlining the purpose of the research, their obligations, and their rights. All subjects provided their informed consent. This study was approved by the Faculty of Social Sciences of the Hellenic Open University, with Ethics Committee approval number 144207, dated 16 October 2023.

Psychological burden

The Depression, Anxiety and Stress Scale - 21 Items (DASS-21) was used to assess psychosocial burden. This questionnaire has been widely used for several years among various populations and age groups and is considered a valid and reliable tool for measuring stress, anxiety, and depression [7-9]. The tool was originally developed by Lovibond and Lovibond [10], and we used the Greek version developed by Lyrakos et al. [11]. The DASS-21 is a self-report questionnaire with 21 questions on a 4-point Likert scale divided into three subscales: stress, anxiety, and depression. Each subscale contains seven questions, and the score for each is calculated by summing the individual items and multiplying the result by two. Higher scores indicate higher stress, anxiety, and depression levels. Based on these scores, five main categories can be distinguished for stress, anxiety, and depression: normal, mild, moderate, severe, and extremely severe [12].

Health-related quality of life

The 36-Item Short Form Survey (SF-36) was used to assess the HRQoL. It is one of the most widely used tools and has been validated and standardized in various languages and clinical conditions [4,13]. It has also been translated and standardized in the Greek language and is considered a valid and reliable tool for assessing the quality of life in the Greek population [14,15]. The questionnaire consists of 36 questions categorized into eight different scales: (1) the Physical Functioning scale (PF, 10 questions), (2) the Role Physical scale (RP, four questions), (3) the Role Emotional scale (RE, three questions), (4) the General Health scale (GH, five questions), (5) the Bodily Pain scale (BP, two questions), (6) the Vitality/Energy scale (VT or Energy, four questions), (7) the Social Functioning scale (SF, two questions), and (8) the Mental Health/Emotional Well-being scale (MH or Emotional Well-being, five questions) [13,16]. Each scale is scored from 0 to 100, with higher scores indicating better health and quality of life [4,13]. Additionally, these eight scales can be grouped into two summary measures of quality of life: the Physical Component Summary (PCS) and the Mental Component Summary (MCS), both of which are also scored from 0 to 100, with higher scores corresponding to better physical and mental health, respectively [4].

Statistical analysis

Statistical analysis involved both descriptive and inferential methods of analysis. Under the descriptive analysis, the frequency, percentage, valid percentage, and cumulative percentage were calculated for all variables. Quantitative variables were analyzed for the mean, standard deviation, range, minimum, and maximum values. However, these variables did not follow a normal distribution, as confirmed by normality analysis (p < 0.05). Hence, non-parametric tests, specifically the Mann-Whitney U test, Kruskal-Wallis H test, and Spearman Correlation, were used for inferential analysis. The chi-square test (x^2^) was also performed to analyze any significant differences in stress, anxiety, depression, and HRQoL based on different demographic and clinical characteristics. All significant differences/associations were evaluated at a 95% confidence interval (p = 0.05). Statistical analysis was performed with IBM SPSS Statistics for Windows, Version 24 (Released 2016; IBM Corp., Armonk, New York, United States).

## Results

Table 1 presents the demographic characteristics of the entire set of participants. Among them, 61.6% (n=93) were female, 37.7% (n=57) were male, and 0.7% (n=1) did not respond. The sample was almost equally divided into groups of children/adolescents and adults. The vast majority of the participants held a secondary or tertiary educational degree. Regarding employment status, nearly half of the participants were students, while one-quarter were employed by private companies. Married individuals accounted for 28.5% of the total (n=43), while the rest were reported to be either single or divorced. Moreover, most participants lived in urban areas.

**Table 1:** Demographic characteristics

Variable	Categories	n	% of total
Gender	Male	57	37.7
	Female	93	61.6
	Missing	1	0.7
Age	Children/adolescents	72	47.7
	Adults	79	52.3
Education	Primary	10	6.6
	Secondary	83	55.0
	Tertiary	46	30.5
	Postgraduate	11	7.3
	Missing	1	0.7
Employment status	Unemployed	11	7.3
	Public sector	13	8.6
	Private sector	36	23.8
	Self-employed	14	9.3
	Retired	1	0.7
	Student	76	50.3
Marital status	Single	94	62.3
	Married	43	28.5
	Divorced	13	8.6
	Missing	1	0.7
Area of residence	Rural	50	33.1
	Urban	98	64.9
	Missing	3	2.0

Table 2 summarizes the clinical characteristics of the participants as a whole. The average child/adolescent had been living with diabetes for the last 6.5 years, while the diagnosis took place at the age of 12.9 years.

**Table 2:** Clinical characteristics based on numerical variables

Variable	n	Min	Max	Mean	Std. dev.
No of years with diabetes	149	1	18	6.5	5.9
Age of diagnosis	148	1	18	12.9	5.2

As illustrated in Table 3, which includes the clinical characteristics of 151 patients, approximately 3 out of 10 patients (n=43) suffered from other chronic illnesses. Furthermore, significant diabetes-related complications were found to have affected 74.2% of the respondents (n=112), with the most common complications being concentration and memory problems (19.2%, n=29), urinary tract infections (9.3%, n=14), retinopathy (7.3%, n=11), gum infections (6%, n=9), ketoacidosis (5.3%, n=8), and vision problems (5.3%, n=8). Most patients followed a special diet (92.7%, n=140) and received insulin therapy (96.7%, n=146).

**Table 3:** Clinical characteristics based on categorical variables

Variable	Categories	n	% of total
Other chronic illnesses	Yes	43	28.5
	No	105	69.5
	Missing	3	2.0
Significant complications due to diabetes (nephropathy, retinopathy, etc.)	Yes	112	74.2
	No	37	24.5
	Missing	2	1.3
Special diet to manage diabetes	Yes	140	92.7
	No	10	6.6
	Missing	1	0.7
Insulin therapy	Yes	146	96.7
	No	4	2.6
	Missing	1	0.7

The results for the entire set of respondents regarding psychological burden are presented in Table 4. The mean score for stress was 16.2 ± 5.8, implying mild stress levels on average. Nevertheless, more than 3 out of 10 (n=46) individuals were found to have moderate stress levels, whereas only 2.7% (n=4) suffered from severe or extremely severe stress. The mean score for anxiety was 10.6 ± 5.6; thus, moderate anxiety levels were reported on average. It is noticeable, though, that 21.8% (n=33) were found to suffer from severe or extremely severe anxiety. In terms of depression, the mean score was also 10.6, with equal standard deviation, but now near the lower bound of mild depression. The main reason for this is that normal and mild depression levels were reported in most cases. In summary, severe and extremely severe levels were captured primarily in the case of the anxiety subscale.

**Table 4:** Results on psychological burden (DASS-21) DASS-21: Depression, Anxiety and Stress Scale - 21 Items.

Subscales (Mean score ± Std. dev.)	Levels (score range)	n	% of total
Stress (16.2 ± 5.8)	Normal (0-14)	27	17.9
	Mild (15-18)	74	49.0
	Moderate (19-25)	46	30.5
	Severe (26-33)	3	2.0
	Extremely severe (≥34)	1	0.7
Anxiety (10.6 ± 5.6)	Normal (0-7)	45	29.8
	Mild (8-9)	15	9.9
	Moderate (10-14)	58	38.4
	Severe (15-19)	26	17.2
	Extremely severe (≥20)	7	4.6
Depression (10.6 ± 5.6)	Normal (0-9)	57	37.7
	Mild (10-13)	43	28.5
	Moderate (14-20)	47	31.1
	Severe (21-27)	3	2.0
	Extremely severe (≥28)	1	0.7

Table 5 presents the SF-36 results for the respondents as a whole. Based on these results, the overall physical health of the participants was considered moderate (50.5 ± 12.7) and slightly better than their overall mental health (44.5 ± 13.2). With respect to physical health, the subscale referring to pain had the highest mean score (65.7 ± 14.7). However, the participants reported low HRQoL in terms of physical role limitations and general health. These two subscales received the lowest mean scores among all the questionnaire items. With respect to mental health, the social functioning and emotional well-being dimensions outperformed the mental component summary, while the emotional role subscale had the lowest mean score among mental health items and the third lowest mean score among all items. Consequently, children/adolescents with type 1 diabetes and their family members were found to have moderate HRQoL; however, three of its components fell short (“general health”, “role physical”, and “role emotional”). 

**Table 5:** Results on HRQoL (SF-36) HRQoL: Health-Related Quality of Life; SF-36:  36-Item Short Form Survey.

Subscales	n	Min	Max	Mean	Std. dev.
Physical functioning	151	10.0	100.0	53.1	18.7
Role physical	151	0.0	100.0	36.8	29.0
Bodily pain	151	22.5	100.0	65.7	14.7
General health	151	5.0	90.0	36.2	15.2
Physical component summary	151	27.5	93.8	50.5	12.7
Role emotional	151	0.0	100.0	37.1	27.9
Vitality/energy	151	5.0	85.0	46.5	12.0
Mental health/emotional well-being	151	4.0	90.0	51.0	12.5
Social functioning	151	25.0	100.0	53.7	13.1
Mental component summary	151	26.0	91.3	44.5	13.2

Tables 6-17 demonstrate the statistically significant differences in psychological burden and HRQoL based on the demographic and clinical attributes of the participants. At first glance, demographics primarily affect the physical health dimension of HRQoL. Age, education level, and employment status appear to have a significant impact on all of its components; younger people, those of lower education level, and students have lower physical health in general. Marital status and area of residence partially affected physical health, with married people and those living in urban areas achieving better physical health. With respect to mental health, only those with postgraduate education reported a higher quality of life. In contrast, more education leads to higher stress. Moreover, men had significantly lower quality of life concerning physical and emotional role limitations but higher vitality. As illustrated in Tables 6-17, clinical attributes were found to affect both psychological burden and HRQoL. The existence of complications due to diabetes seems to play the most critical role, as it affects nearly all the variables. More specifically, the results suggest that minor patients and their families have significantly lower psychological burden (depression, anxiety, and stress) and better HRQoL once no complications due to diabetes occur. In contrast, following a diabetes-specific diet was linked with higher anxiety levels and lower quality of life, negatively impacting physical health. Similarly, those using insulin and those with another chronic illness experienced a negative impact on their mental health and had notably higher stress and depression levels. The saying “time heals all wounds” seems to apply for diabetes, as longer time periods with diabetes were associated with less stress and depression and superior physical functioning. Finally, conflicting results regarding the age of diagnosis and psychological burden and HRQoL emerged. On the one hand, the higher the age at diagnosis of diabetes, the higher the vitality, emotional well-being, and resistance to the psychological burden trio (depression, anxiety, and stress). On the other hand, diagnosis at an older age resulted in lower physical health, especially associated with levels of physical role and general health.

**Table 6:** Significant differences based on gender RP: Role Physical; RE: Role Emotional; VT: Vitality/Energy.

Variables/ Test Statistics	RP	RE	VT
Male	25.0	31.6	48.7
Female	44.1	40.5	45.2
Mann-Whitney U	1,737	2,157	2,122
Wilcoxon W	3,390	3,810	6,493
Z	-3.782	-2.181	-2.126
Asymp. Sig. (2-tailed)	0.000	0.029	0.033

**Table 7:** Significant differences based on age *Correlation is significant at the 0.05 level (2-tailed). **Correlation is significant at the 0.01 level (2-tailed). PF: Physical Functioning; RP: Role Physical; BP: Bodily Pain; GH: General Health; PCS: Physical Component Summary.

Variables/ Test Statistics	PF	RP	BP	GH	PCS
Spearman’s rho	0.239**	0.339**	0.177*	0.399**	0.324**
Sig. (2-tailed)	0.003	0.000	0.030	0.000	0.000

**Table 8:** Significant differences based on the education level S: Stress; PF: Physical Functioning; RP: Role Physical; BP: Bodily Pain; GH: General Health; PCS: Physical Component Summary; VT: Vitality/Energy; SF: Social Functioning; MCS: Mental Component Summary.

Variables/ Test Statistics	S	PF	RP	BP	GH	PCS	VT	SF	MCS
Primary	12.0	53.7	27.5	63.3	33.5	50.2	56.5	63.8	49.5
Secondary	16.5	48.1	29.8	63.3	30.9	46.5	44.6	52.1	41.5
Tertiary	16.8	59.1	46.2	72.7	42.8	56.1	46.3	52.7	45.8
Postgraduate	14.9	65.9	59.1	57.7	51.4	58.6	51.8	61.4	56.9
Chi-Square	8.2	16.6	12.4	21.0	29.7	20.8	10.6	11.1	11.9
df	3	3	3	3	3	3	3	3	3
Asymp. Sig.	0.042	0.001	0.006	0.000	0.000	0.000	0.014	0.011	0.008

**Table 9:** Significant differences based on employment status PF: Physical Functioning; RP: Role Physical; BP: Bodily Pain; GH: General Health; PCS: Physical Component Summary.

Variables/ Test Statistics	PF	RP	BP	GH	PCS
Unemployed	54.5	29.5	66.8	30.0	50.2
Public sector	57.4	44.2	60.8	39.2	50.7
Private sector	61.3	48.6	70.9	46.3	57.5
Self-employed	51.3	42.9	69.3	37.5	52.0
Retired	25.0	100.0	22.5	50.0	49.4
Student	49.1	28.9	63.9	31.4	47.0
Chi-Square	16.5	15.2	13.5	26.5	15.5
df	5	5	5	5	5
Asymp. Sig.	0.005	0.010	0.019	0.000	0.008

**Table 10:** Significant differences based on marital status RP: Role Physical; GH: General Health; PCS: Physical Component Summary.

Variables/ Test Statistics	RP	GH	PCS
Single	29.8	32.5	48.1
Married	52.9	43.5	55.8
Divorced	34.6	39.6	50.9
Chi-Square	19.1	27.0	14.4
df	2	2	2
Asymp. Sig.	0.000	0.000	0.001

**Table 11:** Significant differences based on the area of residence RP: Role Physical; GH: General Health.

Variables/ Test Statistics	RP	GH
Rural	29.5	31.9
Urban	40.3	38.5
Mann-Whitney U	1,875	1,861
Wilcoxon W	3,150	3,136
Z	-2.496	-2.423
Asymp. Sig. (2-tailed)	0.013	0.015

**Table 12:** Significant differences based on the duration of diabetes *Correlation is significant at the 0.05 level (2-tailed). **Correlation is significant at the 0.01 level (2-tailed). S: Stress; D: Depression; PF: Physical Functioning.

Variables/ Test Statistics	S	D	PF
Spearman’s rho	-0.162*	-0.190*	0.221**
Sig. (2-tailed)	0.049	0.020	0.007

**Table 13:** Significant differences based on the age of diagnosis *Correlation is significant at the 0.05 level (2-tailed). **Correlation is significant at the 0.01 level (2-tailed). S: Stress; A: Anxiety; D: Depression; RP: Role Physical; GH: General Health; VT: Vitality/Energy; MH: Mental Health/Emotional Well-being.

Variables/ Test Statistics	S	A	D	RP	GH	VT	MH
Spearman’s rho	-0.316**	-0.211*	-0.316**	-0.205*	-0.183*	0.243**	0.277**
Sig. (2-tailed)	0.000	0.010	0.000	0.012	0.026	0.003	0.001

**Table 14:** Significant differences based on the existence of other chronic illnesses S: Stress; D: Depression; GH: General Health; RE: Role Emotional; VT: Vitality/Energy; MH: Mental Health/Emotional Well-being; SF: Social Functioning; MCS: Mental Component Summary.

Variables/ Test Statistics	S	D	GH	RE	VT	MH	SF	MCS
Yes	18.4	9.8	33.6	29.5	42.9	46.2	49.4	39.7
No	15.2	10.6	37.5	40.6	48.1	53.1	55.6	46.7
Mann-Whitney U	1,639	1,688	1,778	1,804	1,795	1,638	1,802	1,583
Wilcoxon W	7,204	7,253	2,721	2,750	2,741	2,584	2,748	2,529
Z	-2.631	-2.425	-2.066	-2.190	-2.029	-2.645	-2.424	-2.852
Asymp. Sig. (2-tailed)	0.009	0.015	0.039	0.028	0.042	0.008	0.015	0.004

**Table 15:** Significant differences based on the existence of significant complications due to diabetes S: Stress; A: Anxiety; D: Depression; PF: Physical Functioning; RP: Role Physical; GH: General Health; PCS: Physical Component Summary; RE: Role Emotional; VT: Vitality/Energy; MH: Mental Health/Emotional Well-being; SF: Social Functioning; MCS: Mental Component Summary.

Variables/ Test Statistics	S	A	D	PF	RP	GH	PCS	RE	VT	MH	SF	MCS
Yes	16.9	11.4	11.2	47.7	27.7	30.9	46.4	30.4	44.4	48.8	51.3	40.4
No	13.8	8.1	8.9	69.0	60.8	51.6	61.9	55.9	52.2	57.5	61.5	56.6
Mann-Whitney U	1,392	1,310	1,444	882	1,002	588	859	1,306	1,278	1,280	1,645	869
Wilcoxon W	2,095	2,013	2,147	7,210	7,330	6,916	7,187	7,634	7,606	7,608	7,973	7,197
Z	-3.007	-3.377	-2.783	-5.422	-5.043	-6.618	-5.333	-3.853	-3.636	-3.517	-2.386	5.287
Asymp. Sig. (2-tailed)	0.003	0.001	0.005	0.000	0.000	0.000	0.000	0.000	0.000	0.000	0.017	0.000

**Table 16:** Significant differences based on special diet for diabetes management A: Anxiety; PF: Physical Functioning; RP: Role Physical; GH: General Health; PCS: Physical Component Summary; VT: Vitality/Energy; MCS: Mental Component Summary.

Variables/ Test Statistics	A	PF	RP	GH	PCS	VT	MCS
Yes	11.0	50.0	33.0	33.5	48.4	45.6	42.9
No	6.0	93.0	82.5	69.5	75.8	56.0	62.2
Mann-Whitney U	354	33	165	39	62	341	320
Wilcoxon W	409	9,903	10,035	9,909	9,932	10,211	10,190
Z	-2.627	-5.212	-4.322	-5.056	-4.813	-2.819	-2.868
Asymp. Sig. (2-tailed)	0.009	0.000	0.000	0.000	0.000	0.005	0.004

**Table 17:** Significant differences based on the use of insulin therapy S: Stress; D: Depression; RP: Role Physical; RE: Role Emotional; SF: Social Functioning; MCS: Mental Component Summary.

Variables/ Test Statistics	S	D	RP	RE	SF	MCS
Yes	16.5	10.9	34.6	35.4	52.8	43.6
No	8.0	2.5	100.0	83.3	78.1	68.4
Mann-Whitney U	94	50	24	90	102	100
Wilcoxon W	104	60	10,755	10,821	10,833	10,831
Z	-2.331	-2.853	-3.349	-2.709	-2.833	-2.246
Asymp. Sig. (2-tailed)	0.020	0.004	0.001	0.007	0.005	0.025

Finally, Table 18 presents the correlation analysis of psychological burden and HRQoL variables. Stress was positively associated with anxiety and depression and negatively associated with all HRQoL scales, except for role limitations due to physical health. Anxiety was also positively correlated with stress and depression and negatively correlated with quality of life, indicating that higher anxiety levels correspond to increased stress and depression and decreased quality of life. Similarly, depression was positively correlated with stress and anxiety and negatively correlated with all HRQoL scales, suggesting that higher depression levels correspond to higher stress and anxiety and lower HRQoL. Regarding the individual scales and key components of HRQoL, the physical functioning scale showed a statistically significant and positive correlation with all other scales and the two main components, except for the role limitations due to emotional problems scale. The role limitations due to physical health problems scale was significantly and positively correlated with the physical functioning scale, the role limitations due to mental health problems scale, the general health scale, and both components of overall physical and mental health. The role limitations due to emotional problems scale was significantly correlated with all scales and the two main components, except for physical functioning, while the vitality scale also showed statistically significant and positive correlations with all scales and both main components, except for role limitations due to physical health problems. Social functioning was significantly and positively correlated with all scales and both main components, except for role limitations due to physical health problems and bodily pain. The bodily pain scale was significantly correlated with physical functioning, emotional well-being, general health, and both physical and mental health components, whereas general health was significantly correlated with all scales and both main components of overall physical and mental health. Finally, both overall physical and overall mental health were significantly and positively correlated with all quality-of-life scales, as well as with each other.

**Table 18:** Correlations between psychological burden and HRQoL components *Correlation is significant at the 0.05 level (2-tailed). **Correlation is significant at the 0.01 level (2-tailed). S: Stress; A: Anxiety; D: Depression; PF: Physical Functioning; RP: Role Physical; BP: Bodily Pain; GH: General Health; PCS: Physical Component Summary; RE: Role Emotional; VT: Vitality/Energy; MH: Mental Health/Emotional Well-being; SF: Social Functioning; MCS: Mental Component Summary; HRQoL: Health-Related Quality of Life.

Variables/ Spearman’s rho (p-value)	S	A	D	PF	RP	BP	GH	PCS	RE	VT	MH	SF	MCS
S	1	0.687^**^	0.840^**^	-0.352^**^	0	-0.13	-0.167^*^	-0.325^**^	-0.195^*^	-0.660^**^	-0.696^**^	-0.395^**^	-0.452^**^
		(0.000)	(0.000)	(0.000)	(0.999)	(0.111)	(0.040)	(0.000)	(0.016)	(0.000)	(0.000)	(0.000)	(0.000)
A	0.687^**^	1	0.711^**^	-0.471^**^	-0.015	-0.355^**^	-0.291^**^	-0.391^**^	-0.091	-0.508^**^	-0.528^**^	-0.308^**^	-0.354^**^
	(0.000)		(0.000)	(0.000)	(0.855)	(0.000)	(0.000)	(0.000)	(0.266)	(0.000)	(0.000)	(0.000)	(0.000)
D	0.840^**^	0.711^**^	1	-0.425^**^	-0.017	-0.171^*^	-0.217^**^	-0.364^**^	-0.172^*^	-0.651^**^	-0.714^**^	-0.460^**^	-0.455^**^
	(0.000)	(0.000)		(0.000)	(0.833)	(0.035)	(0.007)	(0.000)	(0.035)	(0.000)	(0.000)	(0.000)	(0.000)
PF	-0.352^**^	-0.471^**^	-0.425^**^	1	0.299^**^	0.223^**^	0.492^**^	0.700^**^	0.109	0.421^**^	0.387^**^	0.194^*^	0.346^**^
	(0.000)	(0.000)	(0.000)		(0.000)	(0.006)	(0.000)	(0.000)	(0.185)	(0.000)	(0.000)	(0.017)	(0.000)
RP	0	-0.015	-0.017	0.299^**^	1	0.066	0.604^**^	0.697^**^	0.662^**^	0.088	-0.023	0.098	0.574^**^
	(0.999)	(0.855)	(0.833)	(0.000)		(0.420)	(0.000)	(0.000)	(0.000)	(0.283)	(0.775)	(0.230)	(0.000)
BP	-0.13	-0.355^**^	-0.171^*^	0.223^**^	0.066	1	0.229^**^	0.460^**^	0.074	0.157	0.189^*^	0.159	0.218^**^
	(0.111)	(0.000)	(0.035)	(0.006)	(0.420)		(0.005)	(0.000)	(0.367)	(0.055)	(0.020)	(0.052)	(0.007)
GH	-0.167^*^	-0.291^**^	-0.217^**^	0.492^**^	0.604^**^	0.229^**^	1	0.677^**^	0.429^**^	0.247^**^	0.277^**^	0.192^*^	0.696^**^
	(0.040)	(0.000)	(0.007)	(0.000)	(0.000)	(0.005)		(0.000)	(0.000)	(0.002)	(0.001)	(0.018)	(0.000)
PCS	-0.325^**^	-0.391^**^	-0.364^**^	0.700^**^	0.697^**^	0.460^**^	0.677^**^	1	0.457^**^	0.511^**^	0.394^**^	0.249^**^	0.666^**^
	(0.000)	(0.000)	(0.000)	(0.000)	(0.000)	(0.000)	(0.000)		(0.000)	(0.000)	(0.000)	(0.002)	(0.000)
RE	-0.195^*^	-0.091	-0.172^*^	0.109	0.662^**^	0.074	0.429^**^	0.457^**^	1	0.220^**^	0.234^**^	0.242^**^	0.760^**^
	(0.016)	(0.266)	(0.035)	(0.185)	(0.000)	(0.367)	(0.000)	(0.000)		(0.007)	(0.004)	(0.003)	(0.000)
VT	-0.660^**^	-0.508^**^	-0.651^**^	0.421^**^	0.088	0.157	0.247^**^	0.511^**^	0.220^**^	1	0.780^**^	0.339^**^	0.514^**^
	(0.000)	(0.000)	(0.000)	(0.000)	(0.283)	(0.055)	(0.002)	(0.000)	(0.007)		(0.000)	(0.000)	(0.000)
MH	-0.696^**^	-0.528^**^	-0.714^**^	0.387^**^	-0.023	0.189^*^	0.277^**^	0.394^**^	0.234^**^	0.780^**^	1	0.468^**^	0.585^**^
	(0.000)	(0.000)	(0.000)	(0.000)	(0.775)	(0.020)	(0.001)	(0.000)	(0.004)	(0.000)		(0.000)	(0.000)
SF	-0.395^**^	-0.308^**^	-0.460^**^	0.194^*^	0.098	0.159	0.192^*^	0.249^**^	0.242^**^	0.339^**^	0.468^**^	1	0.431^**^
	(0.000)	(0.000)	(0.000)	(0.017)	(0.230)	(0.052)	(0.018)	(0.002)	(0.003)	(0.000)	(0.000)		(0.000)
MCS	-0.452^**^	-0.354^**^	-0.455^**^	0.346^**^	0.574^**^	0.218^**^	0.696^**^	0.666^**^	0.760^**^	0.514^**^	0.585^**^	0.431^**^	1
	(0.000)	(0.000)	(0.000)	(0.000)	(0.000)	(0.007)	(0.000)	(0.000)	(0.000)	(0.000)	(0.000)	(0.000)	

## Discussion

Regarding psychological burden, this study revealed the occurrence of mild stress (16.16 ± 5.83), moderate anxiety (10.6 ± 5.64), and mild depression (10.6 ± 5.59) levels on average. Moreover, children and adolescents with type 1 diabetes and their family members tend to exhibit psychiatric disorders, with anxiety disorders being the most prevalent. This finding is consistent with the international literature, which indicates that both pediatric diabetes patients and their parents are at a high risk of developing psychosocial disorders [1]. The literature shows that the prevalence of anxiety among parents of children and adolescents with type 1 diabetes ranges from 23% to 55%, while approximately one-third of children and adolescents with diabetes experience anxiety disorders [17-19]. However, in this study, which included both pediatric patients and their parents, only 29.8% of the total sample exhibited normal anxiety levels, indicating a much higher prevalence of anxiety than that reported in the literature. Regarding depression symptoms, the prevalence in the literature ranges from 13% to 26% among children and adolescents with type 1 diabetes and/or their parents [6,17,19-20]. However, in this study, the prevalence of depression, even at mild levels, was particularly high (63.3% of participants). Thus, our study confirms the psychological burden on children/adolescents with type 1 diabetes and their parents. However, significantly higher levels of anxiety and depression were observed compared to those reported in the literature for both children and adolescents with type 1 diabetes and their parents [3,6,17-22]. These differences may stem from the sample collection method, tools used to measure stress, anxiety, and depression, sample composition, and other variations in the research procedures followed across studies.

Regarding HRQoL, participants exhibited moderate levels of overall physical health (50.52 ± 12.66), specifically moderate levels of physical functioning (53.13 ± 18.7), low quality of life regarding physical health limitations (39.76 ± 28.98), moderate-to-good quality of life in terms of bodily pain (65.73 ± 14.67), and low levels of general health (36.19 ± 15.21). The overall mean mental health of participants was moderate to low (44.5 ± 13.25), with low levels of quality of life related to mental health limitations (37.09 ± 27.9), moderate levels of vitality (46.49 ± 12.01), emotional well-being (51 ± 12.46), and social functioning (53.73 ± 13.13). Overall, the quality of life of children/adolescents with type 1 diabetes and their family members was moderate to low. This finding aligns with the international literature, which indicates that both children and adolescents with type 1 diabetes and their parents tend to have lower HRQoL levels owing to the impact of diabetes on their lives [1,3,6,22-24].

Inferential statistical analysis showed that various demographic and clinical characteristics significantly influenced stress, anxiety, depression, and quality of life among children/adolescents with type 1 diabetes and their family members. These influences have also been confirmed in the literature [23]. Factors included gender, where men exhibited lower quality of life regarding role limitations due to physical and emotional problems, but significantly higher vitality than women. The effect of sex on quality-of-life levels has been documented, with the male sex generally associated with a higher quality of life [23,25]. Age was linked to higher quality of life levels regarding physical functioning, role limitations due to physical health, bodily pain, general health, and overall physical health. This partially aligns with the study by Boo et al. [23], but contradicts other findings [25,26]. In contrast to this study, other studies have found no significant relationship between age and HRQoL among children and adolescents with type 1 diabetes [24]. Moreover, participants with higher education levels, who were exclusively parents, exhibited higher stress levels, whereas participants in the primary education category, who were exclusively children with diabetes, exhibited lower stress levels. Thus, parents tend to experience higher stress levels than younger children with type 1 diabetes. This trend is also confirmed by the finding that participants with children exhibited significantly higher stress levels than those without children (i.e., the patients themselves). Participants living in rural areas exhibited significantly lower QoL in terms of role limitations due to physical and general health than those living in urban areas. A longer duration of type 1 diabetes was associated with lower stress and depression levels and higher quality of life. This finding partially aligns with the literature, indicating that the psychological state and quality of life of parents and families improve over time [22,27]. However, this finding contradicts those of other studies linking better quality of life to shorter disease duration [25,26]. Complications due to type 1 diabetes were associated with higher levels of stress, anxiety, and depression, as well as lower quality of life, findings fully supported by international and Greek literature [6,25,26].

Finally, stress, anxiety, and depression were statistically significantly and positively correlated with each other but negatively correlated with HRQoL. Thus, as stress, anxiety, or depression levels increase, quality of life decreases, and vice versa. This connection between anxiety/depression and quality of life in both children and adolescents with type 1 diabetes and their parents has been highlighted multiple times in the literature [3,23,25,28,29], confirming the findings of the present study.

Notwithstanding the several positive implications of our study, there is a major limitation affecting online surveys in general. While there was a reliable sampling frame, the online survey was conducted via email and social media platforms, which means that convenience sampling has occurred to some extent. Consequently, the finding cannot be generalized to the Greek population in the real world. 

## Conclusions

Among children and adolescents with type 1 diabetes and their family members in Greece, high levels of stress, anxiety, and depression are observed, along with moderate levels of quality of life. Stress, anxiety, and depression, as well as quality of life, tend to be significantly influenced by various demographic and clinical factors, such as sex, years since the onset of diabetes, and the presence of diabetes-related complications. Additionally, these factors are significantly interrelated, with stress, anxiety, and depression decreasing as HRQoL improves, and vice versa. Given the importance of HRQoL and anxiety for the mental health of children, the development and implementation of effective interventions aimed at modifying the lifestyles of children and adolescents with type 1 diabetes would be highly beneficial. At the same time, considering the significant psychological burden additionally experienced by the parents of these pediatric patients, intervention by healthcare professionals is deemed necessary. They should pay particular attention to educating the parents of children and adolescents with type 1 diabetes on disease management while also providing continuous psychological support to help them effectively address all challenges arising during diabetes management.

## Appendices

Questionnaire regarding demographic and clinical information of the participants

**Table 19:** Demographic characteristics

Item number	Question	Answers
1	Gender	Male
		Female
2	Age	Please enter an integer
3	Education	Primary
		Secondary
		Tertiary
		Postgraduate
4	Employment status	Unemployed
		Public sector
		Private sector
		Self-employed
		Retired
		Student
5	Marital status	Single
		Married
		Divorced
6	Area of residence	Rural
		Urban

**Table 20:** Clinical characteristics

Item number	Question	Answers
1	No of years with diabetes	Please enter an integer
2	Age of diagnosis	Please enter an integer
3	Other chronic illnesses	Yes
		No
4	Significant complications due to diabetes (nephropathy, retinopathy, etc.)	Yes
		No
5	Specify other chronic illnesses (if any)	If you responded “Yes” to question 4, please specify
6	Special diet to manage diabetes	Yes
		No
7	Insulin therapy	Yes
		No
